# Human Internal Jugular Valve M-mode Ultrasound Characterization

**DOI:** 10.2174/1567202611666140408094014

**Published:** 2013-05

**Authors:** Erica Menegatti, Mirko Tessari, Sergio Gianesini, Maria Elena Vannini, Francesco Sisini, Paolo Zamboni

**Affiliations:** 1 Vascular Diseases Center-University of Ferrara, Programma Fisiopatologia dell'Apparato Vascolare e Day Surgery Az. Osp. di Ferrara, Via Aldo Moro 8, 44124 Cona (FE), Italy; 2 Department of Medical Physics, University of Ferrara, Italy; 3 Department of Morphology, Surgery and Experimental Medicine, University of Ferrara, Italy

**Keywords:** Cerebral venous return, duplex, internal jugular vein, M-mode, vein valves.

## Abstract

In humans the mechanism governing the internal jugular vein (IJV) valve opening and closure is still unclear. M-mode is used in echo-cardiology for the heart valves assessment. Sometimes it was performed also in deep peripheral veins and in vena cava assessment, but never in the IJV valve. Aim of the present study is to investigate the IJV valves physiology in healthy volunteers, by means of both B and M-mode ultrasound.

Eighty-three (83) healthy volunteers (35 Male, 48 Female, 25.7±6.7 y.o.), for a total of 166 IJVs, were enrolled. The entire cohort underwent IJVs high-resolution B and M-mode evaluation, in standardized postural and respiratory conditions. Presence, motility, and number of cusps, as well as their opening and closure mechanism have been assessed.

Bilateral valve absence occurred in 13/83 (16%), whereas at least a one side absence was recorded in 38/83 (46‰ of the cohort) (p<0.0356). Valve leaflets were always mobile and respectively bi-cusps in 34%, or mono-cusp in 27%. The latter was significantly more frequent on the left side (35%) than on the right side (19%) (p<0.0013).

In supine, M-mode valve opening was synchronous with the cardiac cycle.

To the contrary, in an upright position, the valve remained always open and saddled to the wall, independently from the cardiac cycle.

In healthy subjects, the IJV valve leaflets are always mobile, but the significant rate of mono and bilateral absence could suggest a progressive phylogenetic importance loss of such apparatus. M-mode ultrasound enhances the characterization of IJV valve, for this reason it should be taken into consideration to routinely add it to the cerebral venous return investigation.

## INTRODUCTION

In quadrupeds internal jugular vein (IJV) valves are useful to avoid cerebral congestion whenever the head is placed below the heart level, like during feeding [1, 2].

In humans of course there is not the same need, but the mechanisms regulating the IJV valve opening and closure remains unclear. 

Several reports from autoptic or radiological studies demonstrated that the IJV valves present several variants. In a post mortem paper, Lepori [3] found out that in 93% of cases there is a valve in the distal IJV segment, whereas, by means of B-mode ultrasound (US), the same Author demonstrated a valve presence of in only 87% of the investigated subjects.

Only 60% of subjects exhibited a bilateral valve apparatus, so questioning its real utility.

These data were confirmed by Valecchi, finding a valve incompetence in about 90% of people. The same Authors demonstrated how in 75% of the evaluated IJVs the valve was composed by two complete leaflets [4].

**Fig. (1):**
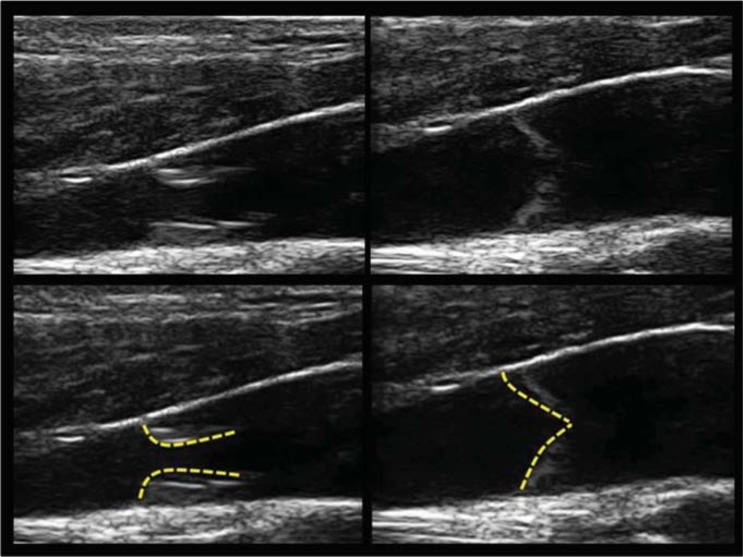
B-mode analysis of the IJV. Top panel: A bicuspid valve with open (left) and close (right) leaflets. Bottom panel: marked valve leaflet in order to better understand the B-mode morphology of valve opening and closure.

In the human cerebral venous outflow regulation, the valve apparatus role is not totally established, yet; especially in its correlation with both the respiration and the posture change [5, 6]. The IJV valve is usually investigated by means of B-mode US. Appropriate morphological data can be obtained by high resolution and frequency probes (Fig. **1**) [7-10].

A deeper valve investigation can be performed thanks to a M-mode scanning, a US software initially developed in echo-cardiography for the heart valves assessment and specifically addressed to the detection of the leaflets movement [11-14].

Recent reports describe the M-mode use to measure diameter variations both of the right ventricle and of the inferior vena cava, in hypovolemic patients [14, 15].

Conversely, other Authors provided an exclusively qualitative assessment of the same anatomical structure, only by B-mode [16].

M-mode was also used in lower limbs venous valves [10], but never in the IJV valve physiology evaluation.

“In M-mode the reflected echoes are converted in a brightness scale and visualized as lines placed sequentially on a time axis (vertical axis= depth, horizontal axis = time, brightness= structure reflectivity). The echoes generated by a transducer plotted over time, form fixed lines or curves when produced by stationary or mobile structures respectively.

This technique has a very high resolution (1-5m sec), which is suitable for studying mobile structures. The valve leaflets in M-mode appear as curved lines when mobile, or as horizontal lines when fixed. The M-mode image does not depend on B-mode image quality, but it depends on tissue acoustic characteristics and on pulse repetition frequency (PRF) of gray-scale image [11, 12].” So a single beam in US scan can be used to produce a M-mode representation, where movement of structures can be depicted as a wavelike shape.

As previously done in other cardiovascular imaging fields [7-12], B-mode information can be implemented by the M-mode analysis. Aim of the present study is to investigate the IJV valves number and mechanism of opening and closure, by means of both high resolution B and M-mode.

## METHODS

Eighty-three (83) healthy volunteers (35 male and 48 female, mean age 25.7±6.7 y.o.), underwent both high resolution B and M-mode IJVs junction evaluations in standardized postural and respiratory conditions [5]. A total of 166 IJVs were assessed. The study was approved by the Local Ethical Committee.

### IJV Valves Investigation

“The investigation was performed in the Vascular Diseases Center of the University of Ferrara, Italy. Environmental conditions were accurately reproduced, always maintaining the same day-time and room temperature (between 1 and 5 p.m.; 23 °C)” [6].

The evaluation was performed in sitting and in supine positions. Moreover, cardiac activity was monitored by a pulse oxymeter (Nellcor™ N−65 Portable Pulse Oximetry Monitor – Covidien China).

“The extracranial veins were assessed by means of an echo-colour-Doppler scanning(ESAOTE MyLab 70, micro-convex array 4.0-7.5 MHz andlinear array 7.5-11 MHz probes).

The exam was performed both by B and M-mode analysis, particularly focusing on the more caudal IJV segment, where the valve is usually located. This region is called J1 and corresponds to the IJV-subclavian vein junction [5, 6]”.

The subject was laid down on a tilt chair, initially in a supine position with the head looking straight ahead. “All the measurements were collected in the longitudinal plane, applying the lightest pressure with the probe [6]”.

**Fig. (2):**
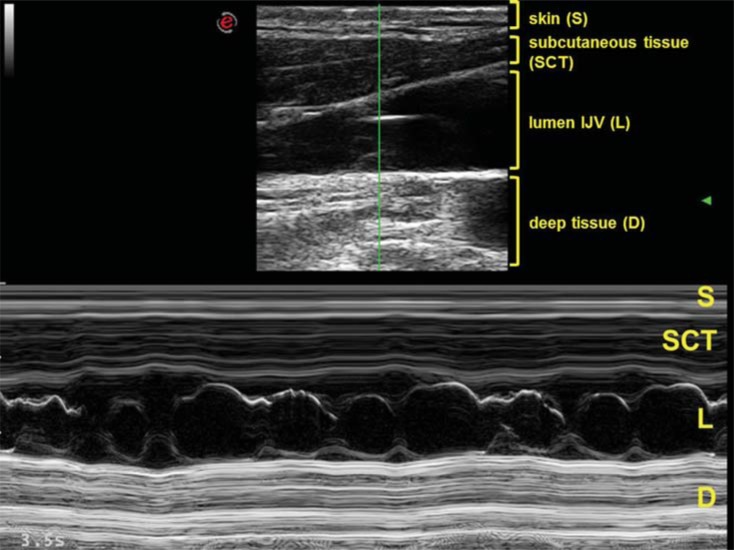
Combined B and M-mode assessment of valve apparatus. Top: B-mode image, with headings in yellow indicating, layer by layer, the corresponding structures. The M-mode sample is visible vertically in the middle of the figure, from the skin surface to the deep tissue of the neck. The M-mode sample passes across the valvular region of the IJV. S= skin; SCT= sub cutaneous tissue; L= IJV lumen; D= deep tissue of the neck. Bottom: corresponding M-mode trace of the bicuspid valve. The movement of the two leaflets are convergent (closure) and divergent (opening) respect to the center of the lumen, drawning a characteristic “8” shape.

The M-mode sample was placed exactly where the valve was previously detected by means of the B-mode (Fig. **2**). In case of valve apparatus was not detectable, the M-mode sample was placed along the entire J1 region in order to confirm the valve absence.

### Assessment of Leaflets Number and Motility

Once the valve was identified in B-mode, the M-mode allowed a significantly more detailed data collection, mainly regarding the leaflets number and their motility. 

The cusps movements were seen along the time axis, so allowing to investigate their opening and closure mechanism in relation respectively to the cardiac cycle and the respiration in both supine and sitting posture (Fig. **2**).

For the former, we used the above described pulse oxymeter, whereas for the latter we adopted a standardized methodology settling a spirometer at 70% of the individual vital capacity [5].

### Statistical Analysis 

Data are presented as mean and standard deviation. Differences were statistically tested for significances by means of two-tailed Fisher Exact Test, and OR 95% CI. Differences < 0.05 were considered significant.

## RESULTS

### IJV Valve Presence Assessment 

Bilateral valve absence occurred in 13/83 patients (16%)both at the B and M-mode analysis, whereas absence at least on one side was recorded in 38/83 (46%) cases (p<0.0356), with not significant differences both between side and gender (Table **1**).

### Leaflets Number Assessment

Table **2** shows the leaflets detected number both on the right and left side, respectively. Particularly, the bi-cusp valve was more frequent on the right side (39/83 cases, 47%), than onthe left side (18/83, 22%). On the contrary, a mono-cusp is more frequent on the left side: 29/83 cases on the left (35%), *vs. *16/83 cases (19%) on the right (p<0.0013). Moreover, differences between gender and side were found to be not statistically significant. Finally, in the whole cohort, a tricuspid valve was never found.

### IJV Valve Motility and M-mode Traces

Table **3** shows the valve cusps M-mode motility, subdivided for gender and side. The assessed valves were all mobile.

At rest, in supine posture, the leaflets motility is mainly modulated by the right atrium contraction following the cardiac rhythm, independently from the respiration. During standardized breathing, we observed that the valve leaflets remain saddled to the vein wall for a long time.

**Fig. (3):**
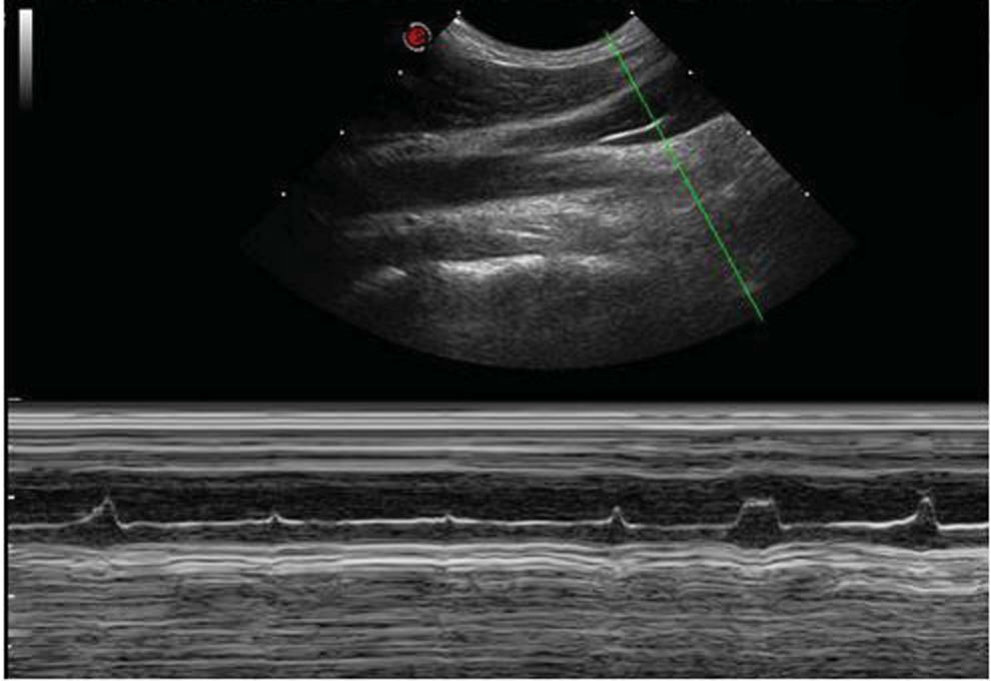
The " Peak" shape. M-mode image of a monocusp valve placed just above the anonymous trunk.A monocuspid trace with a single leaflet,which rhythmically opens and closes entering for a short period of time into the lumen.

**Fig. (4):**
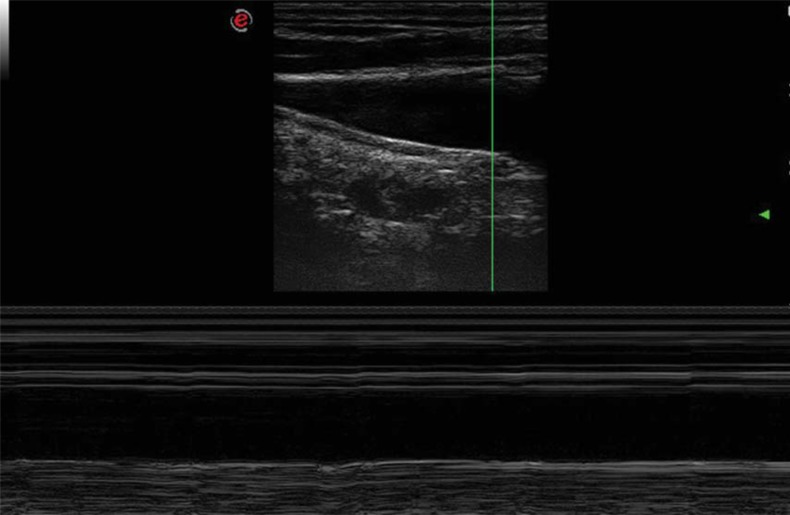
Absence of intraluminal trace. M-mode image of the IJV longitudinal aspect. The M-mode demonstrates the absence of any intraluminal echo signal corresponding to the absence of any valve leaflets.

We found different M-mode traces according to the number of cusps:

1. the "8" shaped (Fig. **2**) bicuspid valve: it’s a cycle of simultaneous opening of two divergent leaflets, followed by a very short time closure, happening when the leaflets become convergent toward the middle of the lumen.
2. The "Peak" shaped (Fig. **3**) mono-cusp valve: it’s the consequence of a rhythmical opening and closure of just one leaflet.
3. The absence of any intraluminal trace corresponds to the absence of any intraluminal echo (Fig. **4**).
4. Finally, in the upright posture the leaflets were always open and well saddled to the vein wall. Closure was not observed in this position, also under a breathing activation at 70% of the individual vital capacity.

## DISCUSSION

Our study pointed out a consistent rate of IJV valve absence, especially on one single side, and slightly greater than the values previously reported in post-mortem and US studies [3, 4, 17, 18].

In our survey the absence of at least one IJV valve apparatus was quite frequent, accounting for 46% of cases, with an unsuspected rate of bilateral absence (16%). 

By comparing our prevalence of visible and mobile valve cusps with those reported in autoptic studies, the valve absence seems to be an overestimated finding. 

For instance, Hamon and Edwards [18] reported 90% of IJV valve in the examined specimen. A possible explanation could be the presence of rudimental valves, very short, thin and practically incorporated into the wall ones. In order to detect this kind of leaflets a high resolution B and M mode are required.

Looking to our data, a progressive phylogenetic loss of IJV valve importance could be hypothesized in humans. This is confirmed by the high prevalence of valve incompetence in other studies [19-22].

Moreover, we highlighted the importance of novel high-tech M-mode informations, an analysis mode that was never used in this kind of investigations previously and that can provide important data concerning both the morphology and the motility of the valve. 

Looking at the morphology, the valve apparatus is more frequently characterized by two leaflets. There is a significant difference in the distribution of monocuspid valves between left and right IJVs. The highest monocusp left sided prevalence could be related to the reduced cross-sectional IJV area respect to the right (data not shown) [23-25].

Conversely to what was occasionally reported in previous studies [17], the tricupid valve morphology was never detected in both our M and B-mode survey. 

Thanks to the never used before M-mode, the main finding in this venous district investigation is that all the assessed leaflets are mobile and always opened and well saddled to the wall, also in the upright posture. 

The M-mode shapes were different according to the cusps number. In case of a bicuspid valve, the M-mode analysis showed a valve commissure aligned to the superior and inferior vessel wall axis (Fig. **1**). Consequently, the leaflets opening and closure is synchronized without overlapping.

The atrium aspiration is the main mechanism governing the rhythmic leaflets movements, being synchronous with the cardiac cycle. The thoracic pump closure time is really brief. To the contrary, the opening time appears to be prolonged by the thoracic pump activation. 

Furthermore, also the posture can influence the valve motility. When the gravitational gradient is favourable, as in up-right posture, [5, 26, 27] the IJV valve remains open for the entire cardiac cycle and the M-mode trace looses its rhythmic movements. 

Nevertheless, our study presents several limitations. The M-mode actually available on US equipment does not permit to measure the time of valve closure. This is fundamental in order to objectify the leaflets kinetics. Moreover, we did not test the competence of the valve by a standardized Valsalva manoeuvre, because this was beyond the aims of the present study. 

Future technical M-mode improvements might also provide better details helping to understand more deeply the IJV valve role as regulatory factor of the cerebral venous return. For instance, Colour M-mode and tissue Doppler imaging may provide further assessment of IJV valve function [13], allowing a technical and more accurate differentiation between normal and pathological, as recently described in Literature [6, 28-31]. Therefore, M-mode propagation along the walls of the IJV pulse by a time-diameter assessment might show differences in wall compliance linked either with circulating blood volume [14, 15] or with altered mechanical properties due to different collagen compositions found in patients with IJV abnormalities [32].

## CONCLUSION

In healthy subjects, the IJV valve leaflets are always mobile, with atrial opening and closure modulation, relatively to the supine position. To the contrary, in upright positions the valve is always open and saddled to the wall.

The significant rate of mono and bilateral absence suggests a progressive loss of phylogenetic importance of such apparatus. In perspective, M-mode could facilitate the IJV valve function assessment in course of a US investigation of the cerebral venous return.

## Figures and Tables

**Table 1.** Rate of presence and absence valve apparatus in IJV.

	Patients	Rate
Bilateral Valve Absence	13/83	16%*
Monolateral Valve Absence	38/83	46%*
Bilateral Valve Presence	32/83	38%*

* p<0.0356

**Table 2.** Morphology of IJV valve apparatus.

Monocusp		Bicusp		Tricusp	
Right	Left	Right	Left	Right	Left
16/83 (9%)	29/83* (35%)	39/83* (47%)	18/83 (22%)	0/83 (0%)	0/83 (0%)

* p<0.0013

**Table 3.** M-mode evaluation of valve motility on right and left side.

	Mobile Leaflets		Not Mobile Leaflets	
	*Right *	*Left *	*Right *	*Left *
*Male *	22/83 (26%)	22/83 (26%)	0/83 (0%)	0/83 (0%)
*Female *	33/83 (40%)	25/83 (30%)	0/83 (0%)	0/83 (0%)
*Total *	55/83 (66%)	47/83 (57%)	0/83 (0%)	0/83 (0%)
